# “Digitally Oriented Materials”: Focus on Lithium Disilicate Ceramics

**DOI:** 10.1155/2016/9840594

**Published:** 2016-08-18

**Authors:** Fernando Zarone, Marco Ferrari, Francesco Guido Mangano, Renato Leone, Roberto Sorrentino

**Affiliations:** ^1^Department of Neurosciences, Reproductive and Odontostomatological Sciences, Division of Fixed Prosthodontics, “Federico II” University of Naples, 80100 Naples, Italy; ^2^Department of Medical Biotechnologies, Division of Fixed Prosthodontics, University of Siena, 53100 Siena, Italy; ^3^Department of Surgical and Morphological Sciences, Dental School, University of Varese, 21100 Varese, Italy

## Abstract

The present paper was aimed at reporting the state of the art about lithium disilicate ceramics. The physical, mechanical, and optical properties of this material were reviewed as well as the manufacturing processes, the results of in vitro and in vivo investigations related to survival and success rates over time, and hints for the clinical indications in the light of the latest literature data. Due to excellent optical properties, high mechanical resistance, restorative versatility, and different manufacturing techniques, lithium disilicate can be considered to date one of the most promising dental materials in Digital Dentistry.

## 1. Introduction

In the last decade, the development of new technologies has moved in parallel with a rapid evolution of restorative materials on the rails of Digital Dentistry, opening new horizons in the field of Prosthodontics. The implementation in the daily practice of the most advanced technologies, like CAD/CAM, laser-sintering/melting, and 3D-printing, has got a synergic impulse from the enhanced mechanical and manufacturing properties of the new generation of dental materials: high strength ceramics, hybrid composites and technopolymers, high precision alloys, and so forth. Among these, metal-free ceramics offer unchallenged advantages like high esthetic potential, astounding optical characteristics, reliable mechanical properties, excellent consistency in terms of precision and accuracy due to the manufacturing technologies, lower costs, and more convenient production timing. In particular, lithium disilicate in the last years has gained maximum popularity in the dental scientific community, offering undeniable advantages.

## 2. Physical-Mechanical Properties and Fabrication Techniques

Lithium disilicate (SiO_2_-Li_2_O) was introduced in the field of glass ceramics in 1998 as a core material, obtained by heat-pressing ingots (Empress 2, Ivoclar Vivadent, Lichtenstein), with a procedure similar to the lost-wax technique used for dental alloys (lithium disilicate heat extrusion at 920°), showing an optimal distribution of the elongated, small, needle-shaped crystals in a glassy matrix with a low number and small dimensions of pores [1]; the core is eventually veneered with fluorapatite-based ceramics, showing noticeable translucency and, at the same time, higher flexural strength (350 MPa) compared to older glass ceramics like the leucite-based ones [2, 3]. Such a material has been discontinued since 2009, replaced in the market by an upgraded typology of lithium disilicate, IPS e.max Press (Ivoclar Vivadent, Schaan, Liechtenstein), in which both the optical and mechanical properties have been enhanced by introducing technical improvements in the production processes [4]. The crystals are smaller and more uniformly distributed; at the same time, this new, more versatile material has introduced the possibility of producing anatomically shaped, monolithic restorations, with no veneering ceramic, just colored on the surface; this innovative indication has become more and more popular in the last years, highly reducing technical complications like chippings and fractures, mainly used for restorations in the posterior areas, where such failures have been shown to be more frequent [5–11]. In order to accommodate the material to the needs of chairside CAD/CAM production processes, another technique has been introduced, based on the use of partially, precrystallized blocks (IPS e.max CAD, Ivoclar Vivadent), containing both 40% lithium metasilicate (Li_2_SiO_3_) crystals and lithium disilicate (Li_2_Si_2_O_5_) crystal nuclei; it is available in different shades and degrees of translucency, depending on the size and density of crystals. In the initial condition, such machineable, bluish blocks show moderate hardness and strength (around 130 MPa); consequently, they are easier to mill, reducing wear of the machining devices at the same time, with evident advantages during chairside procedures [12]. After milling, heat treatment (840–850° for 10 min) determines full crystallization of the material: lithium metasilicates tend to evolve to form lithium disilicates (70%) [13], increasing the flexure strength up to 262 ± 88 MPa [14] with a fracture toughness of 2.5 MPa·m^1/2^ [15]. Compared to the e.max CAD, hot-pressed lithium disilicate exhibits better mechanical properties, like higher flexure strength (440 MPa) and fracture toughness (2.75 MPa·m^1/2^ - IPS e.max Press, Ivoclar Vivadent) [16].

The fabrication processes and machinability affect the restorative quality of monolithic lithium disilicate glass ceramics. A recent investigation analyzed the diamond tool wear, chip control, machining forces, and surface integrity of lithium disilicate after occlusal adjustments. Minimum bur wear but significant chip accumulation was evidenced; furthermore, machining forces were significantly higher than with other glass ceramics. Although the final surface roughness of lithium disilicate was comparable to other glass ceramics, occlusal adjustment caused intergranular and transgranular microcracks, resulting in shear-induced plastic deformations and penetration-induced brittle fractures; such behavior is distinctive of lithium disilicate and very uncommon in other glass ceramics. Consequently, lithium disilicate should be considered the most difficult to machine among glass ceramics for intraoral adjustments [17]. Moreover, thermal processing can influence crystallization kinetics, crystalline microstructure and strength of lithium disilicate restorations. Particularly, extended temperature range (820–840°C versus 750–840°C) and protracted holding time (14 min versus 7 min) produced significantly higher elastic-modulus and hardness properties but showed flexural strength and fracture toughness properties similar to controls (i.e., 750–840°C for 7 min). Rapid growth of lithium disilicates happened when the maximum formation of lithium metasilicates had ended [13].

Recently, innovative fabrication techniques have been proposed to improve the microstructure of lithium disilicate ceramics. Particularly, spark plasma sintering (SPS) was developed specifically for CAD-CAM dental materials. This fabrication process allowed refining the microstructure of lithium disilicate; its densification resulted in textured and fine nanocrystalline microstructures with major lithium disilicate/lithium metasilicate phases and minor lithium orthophosphate and cristobalite/quartz phases [18].

## 3. Mechanical Testing and Fracture Resistance

Due to its intrinsic brittle behavior, lithium disilicate suffers from fatigue failure during clinical service. Microcracks usually initiate in load bearing and/or stress concentration areas, eventually fusing under dynamic loads and creating major flaws that could weaken the lithium disilicate structure; when the ultimate mechanical strength is overcome, catastrophic failures occur [19–22].

Several laboratory studies investigated the fatigue resistance of lithium disilicate single crowns (SCs) and fixed dental prostheses (FDPs) to evaluate experimental designs and testing parameters [20–24]. Different laboratory variables were proved to influence the fatigue resistance of lithium disilicate restorations, such as magnitude of load, number of cycles, abutment and antagonist material, wet environment, and thermocycling; conversely, chewing frequency, lateral movements, and aging technique were considered not influential factors [23]. Single load to fracture after fatigue tests (i.e., combination of dynamic and static loading until fracture) reported highly variable ultimate strength values for this material: from 980.8 N to 4173 N for monolithic SCs and from 390 N to 1713 N for posterior FDPs [23, 24]. Significant comparisons between data were not possible because of the heterogeneity of research designs and testing modalities [24].

Fairly consistent agreement between in vitro and in vivo results was reported. As to SCs, after 2 years of simulated or real service, 100% survival rates were noticed in both laboratory [25] and clinical investigations [26]; in in vitro studies 100% survival rate was reported after 5 years of simulated function as well [20, 27] while the percentage changed to 97.8% in in vivo clinical investigations [26]. Differently, as regards FDPs, the cumulative survival rates at 5 years ranged from 75% to 100% in vitro [28, 29] while the equivalent clinical rate was 78.1% [26]; long-term laboratory investigations simulating more than 10 years of service showed 70% survival rate [30], comparable to the in vivo cumulative survival rate of 70.9% after 10 years of function [26]. The sound level of agreement between in vitro and in vivo data confirmed that laboratory investigations could represent a good simulation of the clinical scenario; nonetheless, this conclusion has to be considered only indicative, since the amount of data is not large enough to indicate consolidated clinical guidelines [24].

A recent systematic review showed significant heterogeneity leading to data inconsistency, because of different study setups and testing parameters. The lack of testing standardization made it almost impossible to perform consistent comparisons between laboratory studies. Consequently, to date, indicative and comparable data about dynamic mechanical testing of lithium disilicate restorations remain still controversial; further investigations with specific standardization criteria are needed [24].

According to in vitro results of dynamic loading, CAD-CAM lithium disilicate SCs should have a thickness of at least 1.5 mm to withstand occlusal loads in posterior areas [22]. Being a filled glass-ceramic, lithium disilicate's final performance as a dental material is strongly related to the type of adhesive cement and accuracy of procedure [31]. To achieve the highest microtensile bond strength (*μ*-TBS) values and best clinical performances, the restorations have to be adhesively luted to the substrates [32, 33]. CAD-CAM monolithic posterior SCs made of lithium disilicate and luted with self-adhesive resin cements showed significantly higher fatigue resistance than feldspathic ceramic restorations. Particularly, lithium disilicate SCs effectively bore the physiological range of masticatory loads, mainly showing repairable fractures. Catastrophic failures were noticed only after load-to-failure tests up to 4500 N [33, 34].

As to implant-supported restorations, although this material showed the highest ultimate strength when compared to feldspathic ceramic and resin nanoceramic onto implant titanium abutments in vitro, no accordance was found between the initial and maximum fracture resistance values of lithium disilicate after chewing simulation with thermocycling simulating 5 years of clinical service [35].

Furthermore, CAD-CAM monolithic lithium disilicate SCs showed an optimum in vitro stiffness and strength values when cemented onto both prefabricated titanium abutments and customized zirconia abutments [36].

## 4. Machinability, Wear Mechanism, and Behavior

Friction and wear effects of lithium disilicate on the opposing natural tooth enamel have been also investigated, with and without fluorapatite coating, showing that they were less severe in unveneered specimens [37]. The initial surface roughness did not influence the final wear but the topography of the wear pattern affected the corresponding wear loss, since a smoother final wear aspect was associated with lower wear. Moreover, superficial wear of lithium disilicate was reported to be sensitive to environmental pH, showing higher friction and wear behavior in basic pH conditions; this was due to the fact that wettability, surface charge, and dissolution trend of lithium disilicate are pH-dependent. The presence of fluorapatite veneering resulted in increased wear of both lithium disilicate crowns and opposing natural teeth; therefore, veneering of the occlusal surface should be avoided.

These results are in agreement with another recent in vitro investigation reporting that zirconia showed less wear than lithium disilicate; in any case, the latter showed occlusal wear equivalent to sound enamel. Enamel wear was reduced after ceramic surface polishing and this supports that this procedure is advisable after performing occlusal adjustments of both lithium disilicate and zirconia restorations. Veneering porcelain significantly increased enamel abrasion; consequently, the use of monolithic zirconia and lithium disilicate should be preferred in areas of strong occlusal contact, in order to limit enamel damage of the opposing teeth over time [38].

After friction against dental enamel, lithium disilicate and monolithic zirconia specimens did not become as rough as feldspathic ceramics. Particularly, when comparing wear effects onto rough, smooth, and glazed surface finishing, eventually rough lithium disilicate became significantly smoother than fine feldspathic porcelain [39].

However, when compared to type III gold, lithium disilicate was more abrasive against human enamel. Enamel opposing lithium disilicate in vitro showed cracks, plow furrows, and surface loss typical of abrasive wear mechanism, resulting in worse wear resistance and friction coefficient than in the presence of antagonist gold [40].

Opposing steatite in chewing simulations, monolithic lithium disilicate yielded higher antagonistic wear and worse wear behavior than monolithic translucent and shaded zirconia, but about half as high as the enamel reference (274.14 *μ*m); particularly, more severe wear patterns on both ceramics and opponents were observed after grinding and glazing [41].

Initial surface finishing and occlusal loads significantly affected the surface roughness, friction, and wear mechanisms of lithium disilicate: as the load increased, surface roughness became more severe and friction coefficient and wear volumes increased in turn. The abrasive wear process can be divided into 2 typologies: 2-body and 3-body abrasive wear. Particularly, in 2-body abrasion wear is caused by hard protuberances on one surface sliding over another while in 3-body abrasion particles are trapped between 2 surfaces but are free to roll and slide. In the presence of smooth lithium disilicate surfaces, 2-body abrasion was dominant while, in case of rough surfaces, 3-body abrasive wear was more significative. Worn lithium disilicate surfaces demonstrated higher sensitivity to delaminations, plastic deformations, and brittle fractures [42].

Two-body wear of lithium disilicate ceramic was found to be comparable to that of human enamel. Furthermore, abrasive toothbrushing significantly reduced gloss and increased roughness of all materials except zirconia [43]. When evaluating mechanical and optical properties, CAD-CAM lithium disilicate glass-ceramic (IPS e.max CAD) demonstrated the most favourable discoloration rate and the lowest 2-body wear on the material side when compared to CAD-CAM composites, hybrid materials, and leucite ceramic; in this study, the wear rate was analyzed in a chewing simulator using human teeth as antagonists [44].

Similarly to other glass ceramics, lithium disilicate can be intraorally repaired in case of chipping. In vitro results using resin composites as restorative materials demonstrated that lithium disilicate can be effectively repaired with hydrofluoric acid etching followed by silanization and adhesive bonding [7, 8, 45].

## 5. Impression Techniques and Accuracy of Fit

Both conventional and digital impression techniques allow for the fabrication of lithium disilicate restorations but the results in terms of marginal accuracy are still controversial [46–51].

An in vitro study reported similar marginal accuracy between conventional and digital impression techniques (112.3 ± 35.3 *μ*m and 89.8 ± 25.4 *μ*m, resp.) and no statistically significant differences were noticed among the different approaches [51]. Differently, the results of a recent in vitro study suggested that pressed and milled lithium disilicate SCs from digital impressions had a better internal fit to the abutment tooth than pressed SCs from polyvinylsiloxane impressions in terms of total volume of internal space, average thickness of internal space, and percentage of internal space at or below 120 *μ*m [50]. Similarly, another in vitro investigation proved that the fully digital workflow provided better margin fit than the conventional fabrication [48]. These results were not in agreement with other investigations demonstrating that the combination of polyvinylsiloxane impressions and Press fabrication techniques for lithium disilicate SCs produced the most accurate 2D and 3D marginal fits [46] and that the combination of digital impressions and pressed lithium disilicate SCs produced the least accurate internal fit [49].

To date, in general, marginal and internal fit of lithium disilicate restorations is significantly influenced by the employed digital impression technique. Although almost all actual digital impression systems show accuracy values within the thresholds of clinical acceptability, significant fit discrepancies are still evident among different digital systems [52].

In vitro microscopical analyses demonstrated that CAD-CAM lithium disilicate SCs had significantly smaller marginal gaps than CAD-CAM anatomic contour zirconia restorations. As to the absolute marginal discrepancy, lithium disilicate SCs showed some overextended margins. Both finish line geometry and fabrication systems significantly influenced the absolute marginal discrepancy [53].

In vivo results by means of the replica technique showed that CAD-CAM lithium disilicate SCs had significantly larger internal axial and occlusal gaps than porcelain-fused-to-metal (PFM) SCs; conversely, marginal gaps were not significantly different. Nevertheless, both PFM and lithium disilicate SCs showed clinically acceptable marginal fit [54]. As regards the restoration adaptation (i.e., marginal and internal fit) of the different manufacturing techniques, evidence is growing that these parameters are more favourable with the hot-pressing technique than with the precrystallized, CAD/CAM milled blocks [46, 55, 56].

## 6. Biocompatibility

Biologic safety of dental ceramics is another main topic on which dental research has been focusing in the last years; such a property can be different even within the same class of materials. Lithium disilicate exhibited more severe in vitro cytotoxicity than dental alloys and composites and became more cytotoxic after polishing [57].

In vitro, human gingival fibroblasts cellular response may reflect variability in soft tissue reaction to different surface materials for prosthetic restorations. In a study by Tetè et al., polished zirconia showed a better integration in respect to the other materials [58]. Analysis on human epithelial tissue cultures, on the other side, demonstrated that lithium disilicate showed the best biocompatibility when compared to zirconia and cobalt-chromium alloys. Consequently, lithium disilicate can be considered a suitable material even for subgingival restorations directly contacting the sulcular epithelial tissues [59]. As to in vivo evidences, the presence of all-ceramic restorations did not induce inflammatory reactions in periodontally healthy patients; no differences between gingival reactions to lithium disilicate and zirconia restorations could be shown [60, 61].

## 7. Clinical Indications and Outcomes

For its outstanding optical properties, mechanical characteristics, ease of processing, and possibility of etching/adhesive bonding, ensuring a minimally invasive approach, lithium disilicate glass ceramics have rapidly become some of the most popular restorative materials in almost all the indications of fixed Prosthodontics [8].

**Figure 1:**
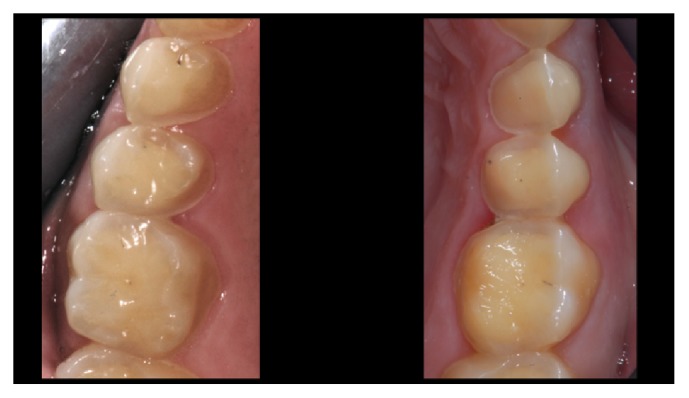
*Case  1* (Monolithic Lithium Disilicate Onlays). Maxillary posterior teeth in a 25-year-old female patient affected by severe food behavior disorder (bulimia). One year before the dental treatment, she was considered healed by a psychotherapist and declared recovered. The teeth were not prepared; only minimal smoothing of some sharp edges was performed.

**Figure 2 fig2:**
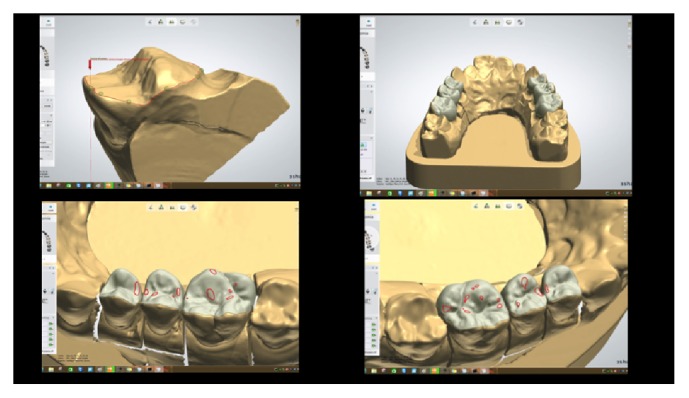
*Case  1* (Monolithic Lithium Disilicate Onlays). After conventional impressions, the casts were scanned by a 3-Shape D700 (3 Shape, Copenhagen, Denmark) digital scanner and analyzed by means of a Dental System  15.5.0 software (3 Shape) and the restorative finish lines were detected. Then, occlusal shape design and contacts were defined.

**Figure 3 fig3:**
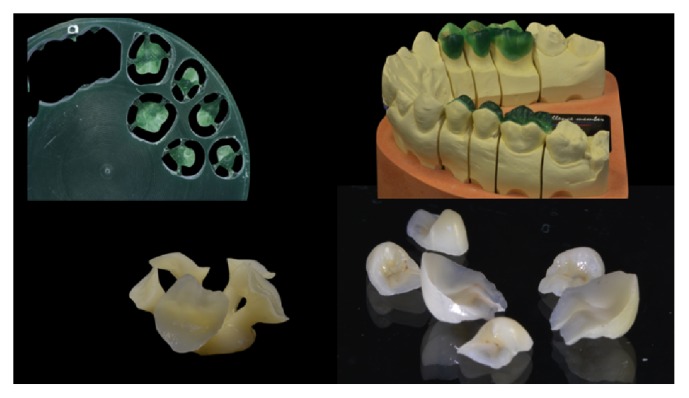
*Case  1* (Monolithic Lithium Disilicate Onlays). The wax patterns of the posterior onlays were milled out of a wax disk (Cera SDD98A18RWC, Sintesi Sud, Avellino, Italy) using a Roland DWX-50 Dental Milling Machine (Whip Mix GmbH, Louisville, KY, USA) and then repositioned on the cast. After careful checking, the lithium disilicate heat pressed onlays (IPS e.max Press MT, Ivoclar Vivadent) were made and eventually polished.

**Figure 4:**
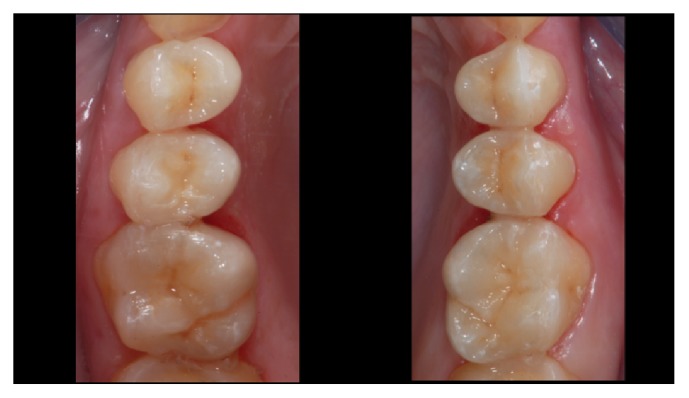
*Case  1* (Monolithic Lithium Disilicate Onlays). The onlays after adhesive cementation.

**Figure 5:**
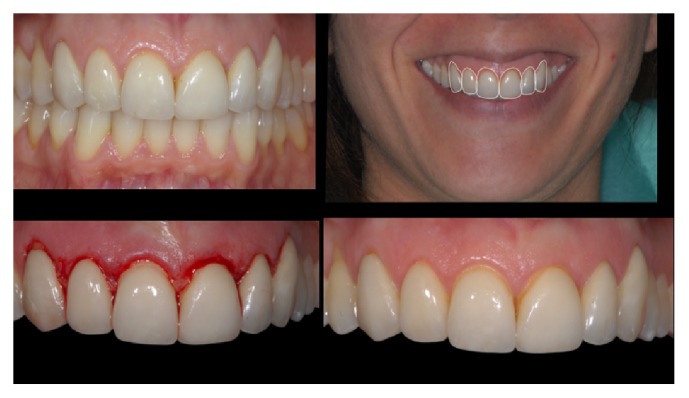
*Case  2* (Bilayered Lithium Disilicate Veneer Replacement). A female patient asked for the replacement of 6 porcelain laminate veneers with discolored and fractured margins. After the study of the case, done with the aid of digital software programs, a crown lengthening procedure was performed.

**Figure 6 fig6:**
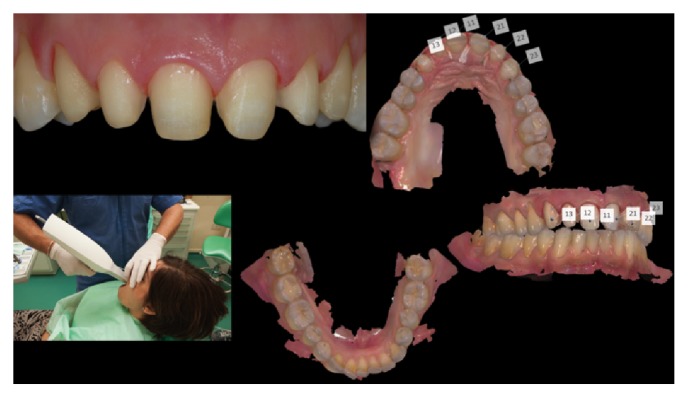
*Case  2* (Bilayered Lithium Disilicate Veneer Replacement). The old veneers were carefully removed under stereomicroscopic control; after the new supragingival preparations, an intraoral scanning device (3-Shape D700) was used to take digital impressions of both dental arches.

**Figure 7 fig7:**
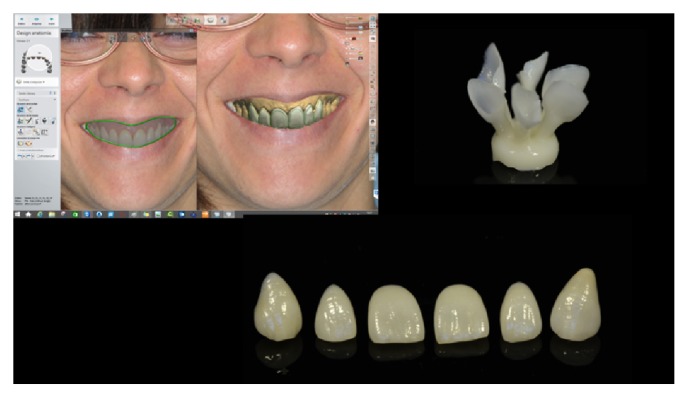
*Case  2* (Bilayered Lithium Disilicate Veneer Replacement). The new smile design was cut away and inserted in the patient's physiognomic image. After designing the new veneers, they were pressed with lithium disilicate (IPS e.max Press MT) and veneered.

**Figure 8 fig8:**
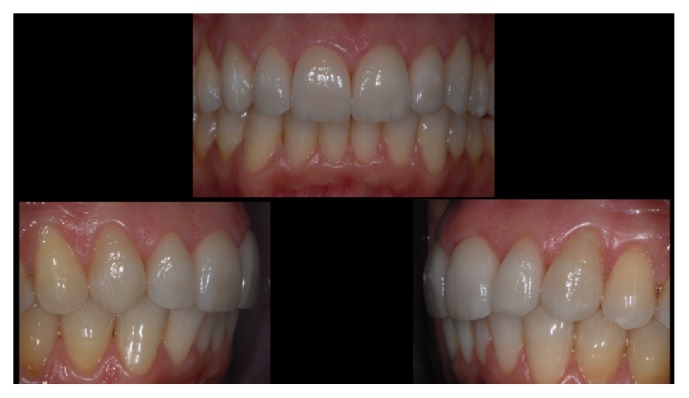
*Case  2* (Bilayered Lithium Disilicate Veneer Replacement). The new veneers at the end of the treatment.

**Figure 9:**
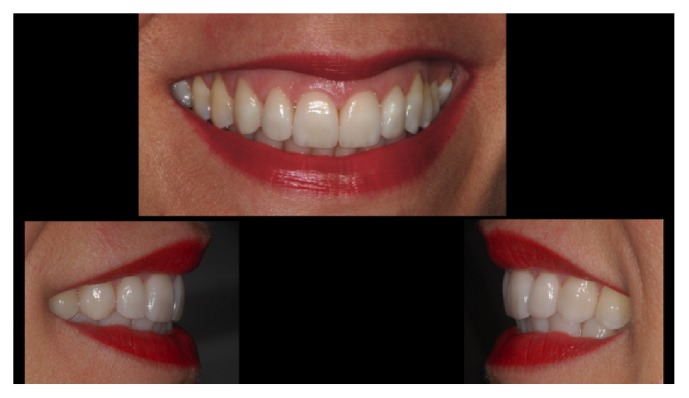
*Case  2* (Bilayered Lithium Disilicate Veneer Replacement). The patient's smile.

Their primary use was addressed for single crowns (SCs). The first clinical studies were conducted on the early typology of lithium disilicate (IPS Empress, Ivoclar Vivadent) and reported quite promising short-term results for the veneered crowns [62, 63]; in particular, Marquardt and Strub, in their prospective clinical trial on both crowns and anterior FDPs, showed for the SCs a survival rate of 100% after 5 years of clinical service [63]. Gehrt et al. [6] analyzed the medium-long term clinical performance of 74 lithium disilicate full-coverage, anterior and posterior crowns after a service time of at least 5 years; all the frameworks, made with the hot-pressing technique from ceramic ingots (IPS e.max Press), were at least 0.8 mm thick and were eventually veneered with a fluorapatite ceramic. The survival rate was 97.4% after 5 years and 94.8% after 8 years of clinical service; among the technical complications, 3 crowns resulted affected by minor chipping. The study revealed that the survival rate was not influenced by cementation type (conventional versus adhesive) or by crown location (anterior versus posterior); on the other hand, in vitro researches have clearly demonstrated that lithium disilicate can bear high stress conditions, like in posterior crowns [64, 65]. Esquivel-Upshaw et al. [66] conducted a 3-year clinical study comparing the performance of veneered lithium disilicate (Empress 2), monolithic lithium disilicate (e-Max Press, glazed), and metal-ceramic crowns (IPS d.SIGN veneer); they observed similar, highly positive results, although a higher degree of surface roughening was detected in the veneered lithium disilicate-based crowns, compared to metal-ceramics, between years 2 and 3. This problem was probably due to degradation/water corrosion of glaze ceramic. Another retrospective, multicentric study on 860 lithium disilicate restorations, both tooth- and implant-supported, including full crowns, laminate veneers, and onlays, reported cumulative survival and success rates beyond 95% for an observational period ranging from 12 to 72 months [8]. The analyzed restorations were both bilayered and monolithic type. More recently, other retrospective studies, with longer observational times, have confirmed low failure rates and very favourable cumulative survival rates with lithium disilicate crowns [65, 67, 68]. Positive clinical outcomes of lithium disilicate reinforced glass ceramics have been confirmed by a recent systematic review [11], showing that 5-year survival rates of all-ceramic SCs made out of lithium disilicate or oxide ceramics (i.e., alumina and zirconia) were similar to the gold standard, metal-ceramic crowns. The widespread diffusion in the daily practice of full-anatomic, monolithic lithium disilicate restorations, characterized by favourable mechanical properties, together with the possibility of manufacturing low thickness restorations adhesively bonded to the dental substrate, has introduced the use of inlays, onlays, and “tabletops” made of this material in the posterior sites, taking advantage of a minimally invasive approach and of a resistant, biocompatible ceramic (Figures 1 [Fig fig2] [Fig fig3]–4). In that research, low fracture rates were reported: 0.91% for monolithic and 1.83% for bilayered single crowns (twice the rate of the monolithic); 4.55% for monolithic FDPs; 1.3% for monolithic; and 1.53% for bilayered veneers (Figures 5 [Fig fig6] [Fig fig7] [Fig fig8]–9). Guess et al. [69] conducted a 7-year prospective “split-mouth” study on both pressed lithium disilicate (IPS e.max Press, Ivoclar Vivadent) and CAD/CAM leucite-reinforced glass-ceramic (ProCAD, Ivoclar Vivadent) partial-coverage restorations. The preparation was performed reducing the entire occlusal surface for a 2 mm thickness, creating a butt joint design at level of the nonsupporting cusps and a rounded shoulder for the supporting cusps. The authors reported high survival rates with both types of restorations, recommending them for a minimally aggressive treatment of extended lesions in posterior teeth. In a recent in vitro research, Sasse et al. [70] advised the need of a lithium disilicate minimum thickness of 0.7–1.0 mm when nonretentive, full-coverage adhesively retained occlusal veneers are used. As regards 3-unit FDPs, according to the manufacturer's recommendations, the use of lithium disilicate should be limited to the replacement of anterior teeth or premolars. Clinical data on this topic is quite controversial. The early, short/medium-term studies, mainly conducted on Empress 2 bilayered lithium disilicate bridges, suggested a certain cautiousness for such an indication: Taskonak and Sertgöz [71] reported a 50% survival rate at 2 years; a prospective clinical trial by Marquardt and Strub showed a fracture rate of 30% after 5 years of clinical service [63]. Makarouna et al. [72], in a randomized controlled trial, after 6 years observed a survival rate of 63% for lithium disilicate FDPs, compared to a much more favourable 95% in the control group (metal-ceramic FDPs).

In a 10-year prospective study conducted by Solá-Ruiz et al. on Empress 2 FDPs, a survival rate of 71.4% was detected, the most frequent complications being postoperative sensitivity, recessions, and marginal discolorations [73]. The introduction of the monolithic, anatomically shaped lithium disilicate FDPs has recently made achieving more favourable outcomes possible.

Some in vitro studies [29, 74, 75] have pointed out that lithium disilicate monolithic crowns and FDPs, both CAD/CAM and hot-pressed, are more resistant to fatigue fracture compared to bilayered, hand veneered ones, showing higher fracture loads (1900 N), that are comparable to the metal-ceramic standard. The lack of the esthetic, weaker veneering material allows a thicker bulk of high strength disilicate; in any case, as regards FDPs, it has to be pointed out that their mechanical performance is multifactorial, being strongly related to many factors, like shape of the structure and size and radius of the connectors among others.

In a long-term prospective study, Kern et al. [5] evaluated the clinical performance of 3-unit, monolithic lithium disilicate FDPs (IPS e.max Press, Ivoclar Vivadent). In this research, the bridges were used not only for the replacement of anterior teeth or premolars (as suggested) but also for missing molars. After 5 years, the survival and success rates were 100% and 91.1%, respectively; after 10 years, they were reduced to 87.9% and 69.8%. Considering that 10-year survival rates of 87.0 to 89.2% have been reported for the “reference” metal-ceramic FDPs by some systematic reviews [11, 76] and that the major, catastrophic failures occurred lately in FDPs replacing missed molars (beyond the manufacturer's recommendations), these evidences advise that the monolithic lithium disilicate can be regarded as a promising candidate to replace metal-ceramics for short-span freestanding bridges.

In the last years, in the light of the concepts of minimal invasivity, economy, and long-term durability, alternative treatment strategies for the anterior single tooth replacement have become more and more popular, taking advantage of the materials' high strength and of the possibility of a reliable adhesive bonding to dental substrates. In particular, cantilevered, all-ceramic, resin-bonded, fixed partial dentures (RBFPD) have been increasingly gaining approval from the dental community, offering a feasible alternative to implant therapy in many cases, particularly when indications for implant therapy are not present, due to general, anatomic, economic, or patient's compliance factors. In such cases, instead of a complete crown, a single veneer adhesively bonded to the lingual side of the support tooth can be used; a careful occlusal check is mandatory, in order to get a proper distribution of stress and a stress limitation on the cantilevered tooth, avoiding lateral and protrusive contacts on the pontic. Also, for this kind of restoration, clinical outcomes are highly encouraging, although data is quite limited to medium-term studies and case series [77–80].

In the last years, the chairside production workflow is gaining more and more interest in the prosthodontic realm, for the speed of delivery and cost reduction of SCs and inlays. The first clinical trials report encouraging results. In the study by Reich and Schierz, besides a survival rate of 96.3% after 4 years, a few biological complications (secondary caries below the crown margin, changing of sensibility perception) and technical complications (need of cervical composite filling) were observed [81].

Recently, Sulaiman et al. [82] have analyzed the clinical outcomes of different IPS e.max lithium disilicate prostheses (SCs, FDPs, veneers, inlays, and onlays), both in the bilayered and monolithic forms, in a 4-year retrospective study on a total of 21.340 restorations. In that research, low fracture rates were reported: 0.91% for monolithic and 1.83% for bilayered single crowns (twice the rate of the monolithic); 4.55% for monolithic FDPs; 1.3% for monolithic and 1.53% for bilayered veneers; and 1.01% for monolithic inlays/onlays. Finally, in the last years, the use of lithium disilicate single crowns bonded onto CAD/CAM zirconia abutments has become increasingly widespread, taking advantage of the high strength and biocompatibility of zirconia, in contact with the peri-implant soft tissues, together with the prosthetic versatility and optical characteristics of lithium disilicate. In vitro studies have demonstrated that these prosthetic solutions exhibit high fracture loads [27, 83] and, at the same time, short-term clinical studies have shown fairly positive outcomes [84], also onto one-piece zirconia implants (Spies). Another clinical approach, also supported by favourable short-term outcomes, makes use of zirconia implant-supported full-arch frameworks (“implant bridges”) on which monolithic lithium disilicate crowns are adhesively bonded [7, 85].

## 8. Conclusions

It is a far from indisputable fact that all of the innovative solutions offered by lithium disilicate are widening the restorative scenario more and more; thanks to the excellent optical properties, the high mechanical resistance, the unique restorative versatility, and the different manufacturing techniques, it is no doubt one of the most promising dental materials in the realm of Digital Dentistry, although more light is still to be shed on some clinical and technical aspects.
